# *Capripoxvirus*-based recombinant vaccine strategies for bluetongue and lumpy skin disease in cattle: current evidence, challenges, and prospects for Central Asia

**DOI:** 10.3389/fvets.2026.1913890

**Published:** 2026-10-22

**Authors:** Alina Kurmasheva, Yerbol Bulatov, Zhanat Amanova, Zhanna Sametova, Dariya Toktyrova, Sholpan Turyskeldy, Ruslan Abitayev, Abdurakhman Ussembay, Dana Mazbayeva, Zhanat Kondibayeva

**Affiliations:** Laboratory for Technologies of Cultivation of Microorganisms, Research Institute for Biological Safety Problems National Holding QazBioPharm, Guardeyskiy, Kazakhstan

**Keywords:** bluetongue, BTV, *Capripoxvirus*, DIVA, LSDV, lumpy skin disease, recombinant vaccine

## Abstract

Lumpy skin disease (LSD) and bluetongue (BT) are transboundary viral diseases affecting livestock in Central Asia. In Kazakhstan, published studies document LSD outbreaks and BTV circulation, although surveillance remains geographically uneven. *Capripoxvirus*-mediated delivery of BTV antigens is not new: studies in small ruminants demonstrated antigen-specific responses but only partial protection. This Mini Review critically evaluates those studies and considers LSDV *Atyrau-5BJN(IL-18)* as a candidate backbone for future investigation. Proposed co-expression of BTV VP2, VP7 and NS3 is an unvalidated research hypothesis. Key uncertainties include serotype matching, each antigen’s contribution to protection, possible interactions among expressed proteins, NS3-based DIVA interference, and recombinant-virus safety and stability.

## Introduction

Lumpy skin disease (LSD) and bluetongue (BT) are transboundary viral diseases of cattle and other ruminants. Their distribution and impact are influenced by animal movements, vector ecology, environmental conditions and viral diversity (1–5). These factors are relevant to Central Asia, where livestock production spans large territories and epidemiological information is uneven across regions (2, 3). LSD was identified in Kazakhstan in 2016 and subsequently affected additional territories (3, 6, 7). Serological and molecular investigations have also reported BTV antibodies and BTV RNA in *Culicoides* spp. in Kazakhstan (2, 8–10). These findings support continued surveillance but do not, by themselves, define the BTV serotypes that should be targeted by a future vaccine.

Vaccination is an important component of LSD and BTV control, but available approaches have different limitations related to safety, duration of immunity, antigenic coverage and surveillance compatibility (11–14). For BTV, the high number of serotypes and the serotype specificity of important neutralizing responses constrain cross-protection (11, 12). Differentiating infected from vaccinated animals (DIVA) is therefore an important consideration when vaccination is used alongside surveillance and movement controls.

*Capripoxviruses* have been investigated as vaccine vectors because their large double-stranded DNA genomes can accommodate foreign genes and their host range is associated with ruminants (15–17). However, the use of *Capripoxvirus* vectors to deliver BTV antigens was demonstrated decades ago (18, 19). The relevant question is therefore not whether this general concept is novel, but whether a locally characterized LSDV backbone and a proposed multi-antigen configuration address a defined research gap. The existing evidence does not establish that the *Atyrau-5BJN(IL-18)* strain expressing BTV antigens would be safe or protective against BTV.

This Mini Review summarizes relevant vaccine platforms, compares foundational *Capripoxvirus*–BTV studies, and examines the rationale and limitations of a proposed LSDV *Atyrau-5BJN(IL-18)* construct expressing VP2, VP7 and NS3. It considers antigen selection, DIVA compatibility, vector biology and biosafety in the epidemiological context of Central Asia.

### Epidemiological context and recent developments

Following LSD outbreaks first reported in Kazakhstan in 2016, vaccination has been used in high-risk areas (3, 6, 7). Vaccination should be integrated with surveillance and movement controls; the relative contribution of each intervention cannot be inferred from outbreak trends alone without appropriate epidemiological analysis.

BTV has a segmented genome comprising ten double-stranded RNA segments and can undergo reassortment. The outer capsid protein VP2 is a principal determinant of serotype-specific neutralizing immunity (11, 12). BTV-9 (western topotype) has been identified in *Culicoides* spp. and clinically healthy livestock in southeastern Kazakhstan (9). This is evidence of regional circulation, but it is not sufficient to select a VP2 sequence for a vaccine intended for broader Central Asian use. Antigen selection would require current, geographically representative molecular surveillance and comparison of VP2 sequences from circulating viruses.

Recent European events illustrate that BTV epidemiology can change over time. BTV-3 emerged in the Netherlands in 2023 and spread to other European countries (20); BTV-12 was reported in Dutch sheep and cattle in 2024 (21). These events reinforce the need for continuing surveillance and careful matching of vaccine antigens to the epidemiologically relevant serotype. They should not be interpreted as direct evidence of the serotypes currently circulating in Central Asia.

### Current vaccine platforms and comparative considerations

No vaccine platform is optimal for every epidemiological and operational setting. Table 1 summarizes selected platforms relevant to LSD and BTV, while recognizing that the evidence base and field experience differ substantially between the two diseases.

**Table 1:** Comparative characteristics of vaccine platforms relevant to LSD and BTV control.

Vaccine platform	Main advantages	Major limitations	DIVA compatibility	Field experience /status	References
Virus-like particles (VLPs)	Native conformational presentation; non-replicating; protection demonstrated in experimental studies.	Assembly and purification complexity; serotype-restricted by VP2.	High with deliberate companion marker assay design.	Experimental for BTV.	(27)
Subunit vaccines	High biological safety; defined antigen composition; flexible design.	Immunogenicity depends on antigen and formulation; adjuvants and booster doses may be needed.	High, provided marker antigen is omitted from vaccine formulation.	Limited/experimental for BTV.	(11, 12)
Adenovirus/MVA vectors	Induces humoral and cellular immunity; non-replicating vectors available.	Pre-existing vector immunity; manufacturing complexity; construct-specific safety.	Potentially compatible depending on expressed transgenes.	Preclinical/early experimental BTV evidence.	(12, 33)
Inactivated BTV vaccines	Established safety profile for licensed products; serotype-specific protection.	Booster doses may be required; protection is serotype-dependent, and duration varies by product and regimen.	Requires companion diagnostic targeting non-expressed proteins (e.g., anti-NS3 ELISA).	Substantial field experience; products deployed against BTV-3 in Europe.	(11, 14, 22, 23)
*Capripoxvirus* vectors	Ruminant-associated vectors; capacity to express heterologous antigens; potential for multi-antigen design.	Unknown genetic stability of multi-cassettes; antivector immunity; vector shedding.	Not intrinsic; depends on heterologous antigen choice (e.g., NS3 interference).	Established for LSDV/SPPV; experimental for BTV delivery.	(15–19, 24, 25, 34)

Inactivated vaccines have substantial practical experience in BTV control and were authorized for use against BTV-3 in Europe during the recent outbreak response (22, 23). Their effectiveness remains serotype-dependent, and regulatory status, target species and timing should be described according to the relevant product documentation. Recombinant platforms may permit flexible antigen design, but their potential advantages must be demonstrated rather than assumed.

### Critical analysis of previous *Capripoxvirus*-BTV studies

Two foundational studies are particularly relevant to the proposed strategy. Their findings establish that *Capripoxvirus*-mediated BTV antigen delivery can induce immune responses, but they do not establish the efficacy of a single vector co-expressing VP2, VP7 and NS3 (Table 2).

**Table 2:** Critical comparison of foundational *Capripoxvirus*–BTV studies and the proposed research hypothesis.

Study/candidate	Antigen and study design	Main findings	Critical interpretation
Wade-Evans et al. (1996) (18)	*Capripoxvirus* expressing BTV-1 VP7; sheep challenged with virulent heterologous BTV-3.	Anti-VP7 antibodies but no detectable neutralizing antibodies before challenge. All vaccinated sheep developed clinical signs; 6/8 recovered, while controls died.	Partial protection against severe clinical outcome; infection was not prevented. Does not establish broad neutralizing protection.
Perrin et al. (2007) (19)	Separate vectors expressing BTV-2 VP2, VP7, NS1 or NS3; sheep (*n* = 11) and goats (*n* = 4). Sheep challenged with virulent BTV-2 3 weeks after immunization.	Seroconversion to VP2, VP7 and NS3 in both species; BTV-specific lymphoproliferation in goats; partial protection in challenged sheep.	Supports immunogenicity and proof of principle, but does not test simultaneous VP2 + VP7 + NS3 expression in one vector or establish efficacy in cattle.
Proposed *Atyrau-5BJN(IL-18)* construct	Proposed co-expression of VP2 + VP7 + NS3; target BTV serotype and VP2 sequence not yet established. No animal study of this construct is reported.	No experimental data on immunogenicity, protection, viraemia, shedding, genetic stability or DIVA performance.	Research hypothesis only. Efficacy or superiority over prior constructs cannot currently be claimed.

Wade-Evans et al. reported a recombinant *Capripoxvirus* expressing VP7 from BTV-1 and evaluated it in sheep (18). Vaccination induced antibodies to VP7 but no detectable neutralizing antibodies against the homologous or heterologous BTV serotype before challenge. After heterologous challenge with virulent BTV-3, all animals developed clinical signs, indicating that infection was not prevented; six of eight vaccinated sheep recovered, whereas the control animals died. The study therefore demonstrated partial protection against severe clinical outcome, not prevention of infection or sterilizing immunity (18).

Perrin et al. evaluated recombinant *Capripoxviruses* expressing individual BTV-2 proteins – VP2, VP7, NS1 or NS3 – in small ruminants (19). Sheep (*n* = 11) and goats (*n* = 4) were immunized. Seroconversion to VP2, VP7 and NS3 was reported in both species, and BTV-specific lymphoproliferative responses were demonstrated in goats. Sheep challenged with virulent BTV-2 three weeks after immunization showed partial protection (19). The study supports the immunogenicity of individual BTV antigens delivered by *Capripoxvirus*, but it does not demonstrate that combining these antigens in one vector improves protection.

Together, these studies provide proof of principle for *Capripoxvirus*-mediated BTV antigen delivery, but show that immune responses do not necessarily prevent infection or confer complete protection (18, 19). Differences in antigen, serotype, animal model and challenge design limit direct comparison. The studies do not establish an optimal antigen combination, the protective value of NS3, or the safety and performance of a multi-antigen construct in cattle.

Accordingly, the contribution of this Mini Review is a critical assessment of the evidence and research gaps associated with investigating the locally characterized *Atyrau-5BJN(IL-18)* backbone in Central Asia. The proposed VP2 + VP7 + NS3 configuration is a hypothesis for testing, not a demonstrated improvement over previous construct. No experimental evidence currently shows that this specific backbone or antigen combination enhances immunogenicity or protection.

### *Atyrau-5BJN(IL-18)* as a candidate recombinant vector

The candidate strain LSDV *Atyrau-5BJN(IL-18)* (GenBank accession ON005067.1) was derived from a field isolate from western Kazakhstan (7). Published descriptions report deletion of LSDV005, LSDV008 and LSDV142, with disruption of the LSDV066 locus by insertion of a cassette encoding bovine interleukin-18 (IL-18) (7, 24, 25). The exact description of each modification should be interpreted alongside the original strain-characterization studies; the genome sequence alone does not demonstrate suitability as a heterologous vaccine vector.

These data do not validate *Atyrau-5BJN(IL-18)* as a heterologous vector. Additional expression cassettes could affect viral fitness, antigen expression, genetic stability, immunogenicity and protection against LSDV. Further assessment would need to address safety, replication, antigen expression, retention of LSDV protection, shedding, transmission and possible effects of IL-18. The cited literature does not establish these properties for the proposed construct; evidence from the parental strain must not be presented as evidence of recombinant safety or efficacy.

### Antigen rationale, immunological uncertainty and DIVA constraints

The proposed antigen set has distinct, but not equivalent, biological rationales. VP2 is a major determinant of serotype-specific neutralizing antibodies, so its sequence would need to be selected using current surveillance and sequence comparison (9, 11, 12, 26). VP7 is a relatively conserved core protein that can induce group-specific antibody responses and may contribute to cellular immune recognition; conservation alone does not establish broad neutralizing activity or protection (18, 27). NS3 is a non-structural protein involved in viral egress and has been investigated as an immunological target, but its contribution to protection when delivered by a *Capripoxvirus* vector remains uncertain (19, 28).

The combined expression of VP2, VP7 and NS3 in one vector has not been experimentally evaluated in the construct proposed here. Co-expression could affect the amount, stability or processing of each antigen and the fitness of the vector. Immunodominance or competition between antigen-specific responses is possible, but its direction and magnitude cannot be predicted confidently from the available evidence. It is therefore not justified to assume that adding VP7 and NS3 to VP2 will improve protection. Pre-existing immunity to *Capripoxvirus* following vaccination or natural exposure could also alter vector replication and the duration or magnitude of heterologous antigen expression; the extent of this effect for this proposed construct is unknown.

DIVA compatibility is a practical design constraint. NS3-based competitive ELISAs have been investigated to distinguish BTV infection from vaccination with vaccines that do not induce anti-NS3 antibodies (29, 30). A recombinant vaccine expressing BTV NS3 could induce antibodies detected by such assays and thereby compromise their discriminatory value. This is a potential diagnostic conflict, not a demonstrated outcome for the proposed construct, because the construct has not been tested. An alternative companion assay targeting a non-vaccine antigen, for example NS1, could be considered, but its performance would require independent development and validation. Because the protective contribution of NS3 is unproven, its inclusion should be evaluated against both immunological objectives and the practical requirement for reliable surveillance.

The safety assessment should distinguish documented properties of existing *Capripoxvirus* vaccines from unresolved risks of the proposed recombinant. No experimental data cited here establish shedding, *Culicoides*-mediated transmission or environmental dissemination of *Atyrau-5BJN(IL-18)*-VP2 + VP7 + NS3. These remain open questions, not established risks or established absences of risk.

- Before any field use could be considered, the candidate would require an evidence-based assessment of genetic stability, retention of attenuation, possible recombination, shedding and transmission, and preservation of protection against LSDV. PCR detection of viral nucleic acid alone cannot establish the presence of infectious virus; where relevant, conclusions about infectious shedding should be supported by appropriate infectivity testing. The scope and sequence of preclinical, field and regulatory evaluations would need to be determined with the competent authorities and applicable standards (25, 31, 32).
- Potential manufacturing or economic benefits, including lower production costs or reduced import dependence, remain hypotheses. They would require comparative evidence on yield, batch consistency, formulation and storage stability, scale-up, quality control and cost-effectiveness. Combining antigens in one vector does not by itself demonstrate lower costs or simpler manufacturing.
- The cited WOAH standards and EMA guidance provide relevant regulatory context for veterinary immunological products (31, 32). The EMA’s 2026 guideline on combined vaccines and associations of immunological veterinary medicinal products remains a draft and should not be described as an adopted final guideline (32). Requirements applicable to a particular live recombinant construct should be confirmed with the current guidance and the competent authority.

In Central Asia, LSD and BTV control should integrate vaccination with molecular surveillance, entomological monitoring and animal-movement management (2, 8–10). Vector distribution and seasonal activity vary by location and species; general statements about climate change should not substitute for regional data. BTV surveillance should identify circulating serotypes and characterize VP2 sequences before antigen selection. Although a recombinant platform might eventually allow antigen updates, the feasibility and regulatory implications have not been demonstrated for this construct. This is best framed as integrated animal-health and ecological surveillance; a broader One Health claim requires a specified human-health dimension.

## Conclusion

LSD and BTV remain important livestock-health concerns in Central Asia, but their vaccine requirements differ. Published studies show that *Capripoxvirus* vectors can deliver BTV antigens and induce immune responses; the available challenge studies demonstrated partial rather than complete protection (18, 19). The locally characterized LSDV *Atyrau-5BJN(IL-18)* strain is a candidate for further investigation, but evidence on the parental strain does not establish the safety, genetic stability or efficacy of a recombinant expressing BTV antigens. In particular, the proposed VP2 + VP7 + NS3 combination has not been experimentally evaluated. Its potential value will depend on surveillance-informed VP2 selection, evidence that each additional antigen contributes meaningfully to protection, assessment of antigen interactions and vector fitness, preservation of LSDV protection, and a workable DIVA strategy. Until such data are available, the construct should be regarded only as a preclinical research hypothesis, not as an established or demonstrably superior vaccine platform.
